# Autosomal STR Profiling and Databanking in Malaysia: Current Status and Future Prospects

**DOI:** 10.3390/genes11101112

**Published:** 2020-09-23

**Authors:** Hashom Mohd Hakim, Hussein Omar Khan, Japareng Lalung, Bryan Raveen Nelson, Geoffrey Keith Chambers, Hisham Atan Edinur

**Affiliations:** 1 DNA Databank Division (D13), Criminal Investigation Department, Royal Malaysian Police, Cheras 43200, Selangor, Malaysia; husseinok@rmp.gov.my; 2 School of Industrial Technology, Universiti Sains Malaysia, Pulau Pinang 11800, Malaysia; japareng@usm.my; 3 Institute of Tropical Biodiversity and Sustainable Development, Universiti Malaysia Terengganu, Kuala Nerus 21030, Terengganu, Malaysia; bryan.nelson@umt.edu.my; 4 School of Biological Sciences, Victoria University of Wellington, P.O. Box 600, Wellington 6140, New Zealand; geoff.chambers@vuw.ac.nz; 5 Forensic Science Programme, School of Health Sciences, Universiti Sains Malaysia, Health Campus, Kubang Kerian 16150, Kelantan, Malaysia; 6 Environmental Futures Research Institute, Griffith University, Nathan, QLD 4111, Australia

**Keywords:** STR genotyping, DNA database, FDDM, criminal investigation, DNA profiling, genetic markers

## Abstract

Science and technology are extensively used in criminal investigation. From the mid- to late-1980s, one of the scientific discoveries that has had a particularly remarkable impact on this field has been the use of highly variable DNA sequence regions (minisatellites) in the human genome for individual identification. The technique was initially referred to as DNA fingerprinting, but is now more widely referred to as DNA profiling. Since then, many new developments have occurred within this area of science. These include the introduction of new genetic markers (microsatellites also known as short tandem repeats/STRs), the use of the polymerase chain reaction for target amplification, the development of DNA databases (databanking), and the advancement and/or improvement of genotyping protocols and technologies. In 2019, we described the progress of DNA profiling and DNA databanking in Malaysia for the first time. This report included information on DNA analysis regulations and legislation, STR genotyping protocols, database management, and accreditation status. Here, we provide an update on the performance of our DNA databank (numbers of DNA profiles and hits) plus the technical issues associated with correctly assigning the weight of evidence for DNA profiles in an ethnically diverse population, and the potential application of rapid DNA testing in the country. A total of 116,534 DNA profiles were obtained and stored in the Forensic DNA Databank of Malaysia (FDDM) by 2019, having increased from 70,570 in 2017. The number of hits increased by more than three-fold in just two years, where 17 and 69 hits between the DNA profiles stored in the FDDM and those from crime scenes, suspects, detainees, drug users, convicts, missing persons, or volunteers were recorded in 2017 and 2019, respectively. Forensic DNA analysis and databanking are thus progressing well in Malaysia and have already contributed to many criminal investigations. However, several other issues are discussed here, including the need for STR population data for uncharacterized population groups, and pilot trials for adopting rapid DNA profiling technology. These aspects should be considered by policy makers and law enforcement agencies in order to increase the reliability and efficiency of DNA profiling in criminal cases and in kinship analysis in Malaysia.

## 1. Introduction

Forensic DNA analysis has been integral to criminal investigations over the last three decades. Worldwide, many developments have seen the introduction of new markers for individual identification, the development of DNA databases, and improved genotyping protocols [1,2,3]. Similar progress has taken place in Malaysia and has been facilitated by the passage of new laws, namely: the DNA Identification Act 2009 (3 September 2009) and the DNA Identification Regulations Act 2012 (30 August 2012) [4]. This legislation allows law enforcement agencies in the country—the Royal Malaysia Police (RMP) and the Department of Chemistry Malaysia (KIMIA)—to legally genotype and store DNA profiles [4]. In Malaysia, DNA profiling is currently limited to the amplification of autosomal short tandem repeat (STR) loci. Admission of DNA profiling results in the courtroom relying heavily on various technical and operational standards [5,6]. In 2019, we described the progress of DNA profiling and DNA databanking in Malaysia for the first time [4]. This report included information on DNA analysis regulations, STR genotyping protocols, chain of custody procedures, database management, DNA sample retention and/or the removal of DNA profiles from the Forensic DNA Databank of Malaysia (FDDM), data sharing between international databases such as the Combined DNA Index System (CODIS) and the United Kingdom National DNA Databank (UKDNAD), proficiency testing, accreditation status, and new markers for human identification. Here, we provide an update on FDDM, focused on the technical issues associated with assigning the weight of evidence for DNA profiles and the potential application of rapid DNA tests in the country.

## 2. Number of DNA Profiles and Hits

The types and numbers of DNA profiles stored in FDDM are shown in Table 1. Beginning in 2012, a total of 116,534 DNA profiles were obtained by 2019, having increased from 70,570 in 2017 [4]. Initially, the AmpFLSTR™ Identifiler™ Direct PCR amplification kit (Applied Biosystems, Foster City, CA) was used by the RMP for genotyping autosomal STR, which was later switched to the GlobalFiler™ Express kit (Thermo Fisher Scientific, Waltham, MA, USA) from 2017 onwards. The GlobalFiler™ Express kit covers an additional eight human genome loci (D22S1045, Y-INDEL, SE33, D10S1248, D1S1656, DYS391, D12S391 and D2S441), compared with only 15 STR loci (D8S1179, D21S11, D7S820, CSF1PO, D3S1358, TH01, D13S317, D16S539, D2S1338, D19S433, vWA, TPOX, D18S51, D5S818, FGA, and a sex-determining marker) for the AmpFLSTR™ Identifiler™ Direct PCR amplification kit. Thus, the STR profiles obtained using the GlobalFiler™ Express kit offer a higher discriminating power. Of particular note is that during this changeover period, only 15,654 new DNA profiles were generated using the AmpFLSTR™ Identifiler™ Direct PCR amplification kit, compared with the 30,310 DNA profiles obtained using the newer GlobalFiler™ Express PCR amplification kit. This is because buccal cell samples from suspects, detainees, drug users, convicted offenders, volunteers, and missing persons were analyzed by the RMP laboratory using the newly adopted GlobalFiler™ Express kit, while the DNA profiles from crime scenes and for paternity cases were genotyped using the AmpFLSTR™ Identifiler™ Direct PCR amplification kit from KIMIA [4]. In our view, KIMIA should urgently consider using the GlobalFiler™ Express kit for generating autosomal STR profiles in order to match their samples with those generated by the RMP laboratory, subsequently increasing the discriminating power of the hit matches.

The system got off to a relatively slow start, and even by 2017, only 17 hits (to the then-existing total of DNA profiles deposited in the FDDM) were obtained. It is important to note that the term “hit” refers to an exact (100%) concordance between two DNA profiles stored in the FDDM and those from crime scenes, suspects, detainees, drug users, convicted offenders, missing persons, or volunteers. This number increased to 69 by 2019 (Table 2 and Table S1; refer to Hakim et al. [7] for the definition of violent crime in Malaysia). The number of hits thus increased by more than three-fold in just two years, and this observation reflects the greatly increased number of DNA profiles deposited in FDDM over recent years. Similar patterns have been reported for other well-established national DNA databases, such as those in Austria and Germany. Their repositories store large amounts of DNA profiles and have seen high rates of profile hits since 1997 and 1998, respectively [6]. The Austrian DNA database contains 279,739 entries and 32,765 hits, while Germany’s DNA database stores 1,133,973 DNA profiles with 209,308 hits, all recorded up until the end of December 2015 [6]. Therefore, FDDM has the potential to increase its rate of hits because of the increasing number of DNA profiles stored in the database that continue to accumulate. In addition, the hit rate also depends on the number of years the database has been in operation as well as the source of profiles (discussed below) in the database. These factors should be considered by FDDM in order to increase the benefits of having a DNA databank in the country.

The major entry categories in FDDM were those DNA profiles obtained from suspects and convicted individuals (Table 1). These two groups also contributed the highest number of hits. The DNA profiles listed in Table 1 were obtained only from ~30% of convicted individuals. In our opinion, the number of hits against this index would increase significantly if a larger portion of convicted individuals were genotyped and their DNA profiles were stored in FDDM. However, in addition to cost and labor issues, this effort will have a contingent time constraint, because the DNA profiling of convicted individuals is limited by the length of the sentence that they have to serve in prison, even though it is allowed, by law, to genotype and store DNA profiles from individuals soon after they are found guilty [4]. These activities are regulated by both the DNA Identification Act of 2009 and the DNA Identification Regulations of 2012. Together, they allow for genetic materials to be collected from convicted felons, crime scenes, suspects, detainees, volunteers, and drug users, as well as samples from missing persons cases.

## 3. DNA Population Data for Casework and Paternity Testing Statistics

It is important to recognize that Malaysia is a heterogeneous multi-ethnic country populated by people with many different genetic lineages. These include three Orang Asli groups (Semang, Senoi, and Proto-Malays), Malays (including Malay sub-ethnic groups), Chinese, and Indians. All groups are descendants of ancestrally- and genetically-unrelated ethnic groups [8,9,10]. Malays, Proto-Malays, and Malay sub-ethnic groups might be considered as exceptions, because they are ancestrally-related Austronesian speakers [11,12,13,14]. However, there is evidence of genetic dissimilarities between them, arising largely from founder effects, gene flow, and natural selection [15,16,17,18]. Therefore, probative values (e.g., the power of exclusion and discrimination for paternity and criminal cases) inferred from STR data for just one particular population group cannot be used when calculating the weight of the DNA evidence for the general Malaysian population, even among the three historically-related population groups highlighted above. The STR population datasets currently available for various population groups in Malaysia are listed in Table 3. These datasets were mainly produced by researchers from academic institutions [8,9,19,20,21,22,23,24,25,26,27,28,29,30,31,32,33,34,35], and are not part of the RMP database. Mathematical probabilities calculated from these data do indicate that the STR loci tested are reliable for the identification of individuals in criminal cases and for kinship analysis associated with paternity disputes and immigration applications. However, STR population data for many other population groups have never been reported, including for Proto-Malays and Senoi in Peninsular Malaysia, as well as other ethnic groups of Sabah (Murut, Lundayeh, and Suluk) and Sarawak (Orang Ulu, Tagal, and Punan Bah) in Borneo. This may lead to technical challenges when submitting DNA test results into evidence before the court when no appropriate STR population dataset for calculating match probabilities is currently available [36]. Previously, we also proposed the inclusion of other genetic markers, such as mitochondrial DNA (mtDNA) and Y-chromosome STR (Y-STR), which have several advantages over autosomal STR in paternity disputes, kinship analyses, missing persons identification, and sexual assault cases [4]. Similarly, appropriate population data are needed before these two markers can be added as part of the DNA profiling procedures in Malaysia. Our survey using academic search engines such as Google Scholar and Scopus showed that the Y-STR [37,38,39,40,41,42] and mtDNA [43,44,45,46,47,48,49,50,51,52] population data are also lacking for many population groups in Malaysia (refer to Tables S2 and S3). Therefore, funding should be allocated urgently, not only for the establishment of mtDNA and Y-STR genotyping methodologies in RMP and KIMIA laboratories [4], but also for the development of autosomal STR, Y-STR, and mtDNA population databases for uncharacterized population groups in Malaysia.

## 4. Rapid DNA Testing

One major advantage of forensic DNA analysis is the power of the evidence to link or exclude individuals from a particular crime scene. However, conventional DNA profiling is a lengthy process, and it may take an especially prolonged time for laboratories that receive high volumes of casework, coupled with limited numbers of analysts and restricted genotyping facilities.

Several companies have developed rapid DNA test machines (e.g., ANDE™ Rapid DNA Analysis System developed by Accelerated Nuclear DNA Equipment, and Applied Biosystems RapidHIT^®^ ID system by Thermo Fisher Scientific Inc., Waltham, MA, USA) where DNA profiles can be generated in a much shorter time than what is possible using the conventional profiling methodology. In addition, these new DNA profiling platforms are fully automated, and only require a sample injection from swabs to produce STR profiles ready for matching against those already stored in the national DNA databank [53,54]. This means that DNA profiling can now be performed outside of accredited laboratories and by operatives (e.g., police officers or civilian employees) that have only received basic training on the instruments. These rapid DNA tests are currently limited to buccal cell samples taken from willing individuals, although the potential use of rapid DNA testing on casework samples has been explored by Hares et al. [55]. Standard laboratory analyses and specialized techniques are still essential to profile STR from crime scene samples, as these may vary in type, quality, and quantity. They may also be partially degraded or contain mixtures of DNA from several different individuals [56].

From a practical perspective, the adoption of rapid DNA testing will be cost-effective in terms of police time saved through the rapid exclusion of detained innocent suspects. This is not to mention the value of relieving pressure on suspected innocent persons and their families, because turnaround time for rapid DNA testing is shorter than for conventional DNA profiling methodology. Therefore, the rapid DNA test could be particularly valuable to law enforcement agencies, including in Malaysia. In current practice, the buccal cell samples from convicted persons, suspects, detainees, volunteers, and drug users, as well as all of those associated with missing persons cases, are collected at district and state police stations throughout the country, but are analyzed by the RMP DNA laboratory in Cheras, Selangor [4]. This agency also manages the national DNA databank. The necessary transfers of casework material can cause delays in criminal investigations, because the buccal samples need to be delivered to the RMP DNA laboratory. This may take up to 1–3 days, depending on the logistical arrangements and conditions. In contrast, a rapid test DNA profile can, in principle, be obtained quickly and easily early in the booking process if a rapid DNA platform is located at each district police station. Rapid DNA testing can also be adopted for paternity testing and kinship analyses. Currently, DNA profiling for paternity cases and kinship analyses in Malaysia is conducted on a case-by-case basis by KIMIA, using either blood or buccal cell samples. KIMIA DNA laboratories use the conventional STR profiling technology (i.e., AmpFLSTR^®^ Identifiler^®^ PCR kit) for the genotyping of autosomal STR loci, and the results are only released 25 days after case registration [57]. Therefore, the introduction of rapid DNA testing for paternity cases and kinship analyses at KIMIA would obviously reduce the workload of the staff, and DNA results can be returned to clients in a shorter period of time.

There is strong incentive to investigate rapid DNA profiling techniques, given that they have several advantages compared with conventional DNA methods for the STR profiling of buccal swab reference samples currently adopted by the law enforcement agencies in Malaysia. However, several technical and operational standards will need to be considered before rapid DNA methodology can be thoroughly evaluated and considered for use in the country.

### 4.1. DNA Profiling Regulations

All DNA profiling work in Malaysia is based on rules established in the DNA Identification Act of 2009 and the DNA Identification Regulations Act of 2012. This legislation only allows DNA to be analyzed by experts who have been vetted by the Minister of Home Affairs, and their work should be conducted in RMP and KIMIA facilities. In this context, amendments will need to be passed into law so that rapid DNA tests can be conducted outside these premises by trained and competent individuals, and should go ahead as soon as possible if the pilot testing scheme proves to be successful.

### 4.2. Pilot Study

We recommend a pilot study to test the validity and reliability of the available rapid DNA machines and supporting systems before the technology can be rolled out nationwide (refer to the following subsections). The study should be designed to evaluate the overall work flow of the rapid DNA testing methodology, including DNA profiling at booking stations, data processing, and returning hit results from the FDDM. The pilot study may involve several selected booking stations in the country and provide general estimates of costs involved in training and instrumentation, as well as any other technical issues related to data management and sharing. In some cases, pilot tests may need to be repeated if major improvements were recommended during the original testing scheme.

### 4.3. Supporting Unit

If rapid DNA testing technology proves successful, and plans and legislation are put in place for its introduction at local centers, then further developments will be necessary. In particular, information technology facilities will need to be updated, extended, and made appropriate for the new and extended tasks at hand. This is because DNA profiles from booking stations will need to be verified and uploaded to FDDM for matching. When doing this, several restrictions and appropriate control measures should be set in place beforehand in order to prevent otherwise private DNA information being leaked from FDDM, and/or access to FDDM data by unauthorized personnel. This safeguard can perhaps be best achieved by setting up an electronic operator-controlled call center at the FDDM office, with the main task of receiving DNA profiles and returning hit results to booking stations.

### 4.4. Staffing and Funding

A systematic national-scale plan will be needed for the implementation of rapid DNA testing. Ideally, rapid DNA machines and trained personnel will be located at each booking station. However, this will be very costly and may not really be practical for those stations located in low crime rate areas. Therefore, the police force might need to recruit new staff or train existing personnel, and adjust the number of booking stations incrementally according to where there is a need for facilities equipped with a rapid DNA testing capability. This strategy will help to maximize the benefits of rapid DNA test implementation and minimize costs if it is to be implemented throughout the country, including in booking stations with a low demand/much lower crime rates. Thus, staffing and funding are the critical elements to be considered. Improper planning and management will not only affect the potential application of rapid DNA testing in the country, but also affect other technical and operational aspects that need to be maintained and implemented. These include those discussed earlier by Hakim et al. [4], such as accreditation, capacity building, infrastructure development, and the adoption of new markers and the latest technology.

## 5. Conclusions

Overall, forensic DNA analysis is now progressing well in Malaysia and has already contributed to many criminal investigations. However, several issues discussed here should be considered by policy makers and law enforcement agencies in order to increase the reliability and efficiency of DNA profiling in criminal cases and kinship analysis. These include increasing the proportion of DNA profiles taken from convicted individuals, developing STR population data for the remaining uncharacterized population groups in Malaysia, and running pilot trials to evaluate the reliability and practicality of adopting rapid DNA profiling technology.

## Figures and Tables

**Table 1:** Types and numbers of DNA profiles stored in the Forensic DNA Databank of Malaysia (FDDM) for 2017 and 2019.

	Accumulated Data up to 2017 ^#^		Accumulated Data up to 2019	
DNA Database Entry Classes	AmpFLSTR™ Identifiler™ Direct PCR Amplification Kit (16 Loci)	GlobalFiler™ Express PCR Amplification Kit (24 Loci)	AmpFLSTR^™^ Identifiler™ Direct PCR Amplification Kit (16 Loci)	GlobalFiler™ Express PCR Amplification Kit (24 Loci)
Crime scene *	396	0	8420	0
Suspect	22,792	36	27,451	21,835
Detainees	183	28	183	379
Drug users	6506	3234	6506	3975
Convicted	24,600	7803	24,600	14,954
Volunteer	4786	42	7757	189
Missing person	3	161	3	282
Total	59,266	11,304	74,920	41,614

^#^: Number of short tandem repeat (STR) profiles obtained using Identifiler™ Direct and GlobalFiler^TM^ express kits as reported by Hakim et al. [4]; *: includes the profiles from dead bodies and trace samples from the victim in order to ID the perpetrator. Refer to Hakim et al. [4] for details of the database entry classes. Source: data were obtained with permission from the DNA Databank Division (D13), Criminal Investigation Department, Royal Malaysia Police.

**Table 2:** List of hits from FDDM by year.

Year	Type of Offense/Case	Number of Hits	Total Number of Hit
2012	Rape	1	1
2013	Murder	1	1
2015	Robbery/rape	1	3
	Gang-robbery	2	
2016	Rape	4	6
	Robbery/rape	1	
	Housebreaking	1	
2017	Rape	1	6
	Possession of an unlawful firearm	1	
	Gang-robbery	1	
	Murder	2	
	Identification of unidentified body	1	
2018	Rape	1	20
	Housebreaking	14	
	Gang-robbery	4	
	Murder	1	
2019	Theft vehicle	1	30
	Rape	1	
	Robbery/rape	4	
	Housebreaking	10	
	Gang-robbery	6	
	Murder	2	
	Possession of an unlawful firearm	2	
	Identification of unidentified body	2	
	Custody/control dangerous drugs	1	
	Carnal intercourse	1	
End January 2020	Housebreaking	1	2
	Identification of unidentified body	1	
	Total hits		69

Source: data were obtained with permission from the DNA Databank Division (D13), Criminal Investigation Department, Royal Malaysia Police.

**Table 3:** List of autosomal STR studies in various population groups in Malaysia.

Region	Ethnicity	Sample Size (n)	Number of STR Loci	Panel Used for Amplification	Reference
Peninsular Malaysia (main ethnic group)	Malay	210	15	AmpFLSTR^®^ Identifiler^®^ PCR kit (Applied Biosystems)	[8]
		100	10	Promega Geneprint™ STR System (Promega)	[19]
		185	15	Promega PowerPlex™ 16 System (Promega)	[22]
		110	15	AmpFLSTR^®^ Identifiler^®^ PCR kit (Applied Biosystems)	[26]
		341	15	AmpFLSTR^®^ Identifiler^®^ PCR kit (Applied Biosystems)	[32]
	Chinese	219	15	AmpFLSTR^®^ Identifiler^®^ PCR kit (Applied Biosystems)	[8]
		102	9	Promega Geneprint™ STR System (Promega)	[20]
		216	15	Promega PowerPlex™ 16 System (Promega)	[22]
	Indian	209	15	AmpFLSTR^®^ Identifiler^®^ PCR kit (Applied Biosystems)	[8]
		102	9	Promega Geneprint™ STR System (Promega)	[21]
		195	15	Promega PowerPlex™ 16 System (Promega)	[22]
	Sikh	109	9	Promega Geneprint™ STR System (Promega)	[24]
	Gurkha	100	9	Promega Geneprint™ STR System (Promega)	[25]
Peninsular Malaysia (Malay sub-ethnic group)	Acheh	7	15	AmpFLSTR^®^ Identifiler^®^ PCR kit (Applied Biosystems)	[31]
	Champa	13	15		
	Rawa	11	15		
	Kedah	9	15		
	Minangkabau	23	15		
	Bugis	15	15		
	Kelantan	43	15		
	Banjar	18	15		
	Javanese	135	16	Promega PowerPlex™ 16 System (Promega)	[23]
		14	15	AmpFLSTR^®^ Identifiler^®^ PCR kit (Applied Biosystems)	[31]
East Malaysia, Borneo (Sabah ethnic group)	Kadazan-Dusun	154	15	Promega PowerPlex™ 16 System (Promega)	[28]
		271	15	Promega PowerPlex™ 16 System (Promega)	[29]
		100	3	Promega Geneprint™ STR System (Promega)	[33]
	Bajau	159	15	Promega PowerPlex™ 16 System (Promega)	[29]
	Rungus	209	15	Promega PowerPlex™ 16 System (Promega)	[29]
	Kedayan	200	21	GlobalFiler™ Express PCR Amplification kit (Applied Biosystems)	[34,35]
East Malaysia, Borneo (Sarawak ethnic group)	Iban	195	15	AmpFLSTR^®^ Identifiler^®^ PCR kit (Applied Biosystems)	[9]
		100	3	Promega Geneprint™ STR System (Promega)	[30]
	Bidayuh	195	15	AmpFLSTR^®^ Identifiler^®^ PCR kit (Applied Biosystems)	[9]
	Melanau	128	15	AmpFLSTR^®^ Identifiler^®^ PCR kit (Applied Biosystems)	[9]
Northern Peninsular Malaysia, Orang Asli (Negrito sub-tribe group)	Jahai	30	7	ABGene Ready Mix (Thermo Fisher Scientific)	[27]
	Bateq	18	7		[27]
	Mendriq	14	7		[27]

## Supplementary Materials

The following are available online at https://www.mdpi.com/2073-4425/11/10/1112/s1, Table S1. List of hits from FDDM by type of offences; Table S2. List of Y-STR studies in various population groups in Malaysia; Table S3. List of mtDNA studies in various population groups in Malaysia.

## Abbreviations

DNA	Deoxyribonucleic acid
STR	Short tandem repeats
FDDM	Forensic DNA Databank of Malaysia
PCR	Polymerase chain reaction
CODIS	Combined DNA Index System
RMP	Royal Malaysia Police
UK NDNAD	United Kingdom National DNA Databank
