# Supplementary material for: Autosomal STR Profiling and Databanking in Malaysia: Current Status and Future Prospects
Source: Genes (Basel). 2020 Sep 23;11(10):1112. doi: 10.3390/genes11101112 (PMC7597947; doi:10.3390/genes11101112)
Supplement: Supplementary file 1 [file genes-11-01112-s001.pdf]

Table S1. List of hits from FDDM by type of offences.

No.	Type of offense	Hit indices	Hit year
1	Theft vehicle	Convicted vs. crime scene	Mac 2019
2	Rape	Convicted vs. crime scene	Feb 2017
3	Rape	Suspect vs. crime scene	May 2012
4	Rape	Suspect vs. crime scene	Jan 2016
5	Rape	Suspect vs. crime scene	July 2016
6	Rape	Convicted vs. crime scene	Feb 2016
7	Rape	Convicted vs. crime scene	Apr 2018
8	Rape	Convicted vs. crime scene	July 2016
9	Rape	Convicted vs. crime scene	Feb 2019
10	Robbery / Rape	Volunteer vs. crime scene	Apr 2019
11	Robbery / Rape	Convicted vs. crime scene	Oct 2015
12	Robbery / Rape	Crime scene vs. crime scene	Sept 2016
13	Robbery / Rape	Crime scene vs. crime scene	May 2019
14	Robbery / Rape	Convicted vs. crime scene	Sept 2019
15	Robbery / Rape	Convicted vs. crime scene	Nov 2019
16	Housebreaking	Convicted vs. crime scene	July 2016
17	Housebreaking	Suspect vs. crime scene	May 2018
18	Housebreaking	Suspect vs. crime scene	July 2018
19	Housebreaking	Suspect vs. crime scene	Sept 2018
20	Housebreaking	Suspect vs. crime scene	Sept 2018
21	Housebreaking	Suspect vs. crime scene	Sept 2018
22	Housebreaking	Suspect vs. crime scene	Sept 2018
23	Housebreaking	Suspect vs. crime scene	Sept 2018
24	Housebreaking	Suspect vs. crime scene	Oct 2018
25	Housebreaking	Suspect vs. crime scene	Oct 2018
26	Housebreaking	Suspect vs. crime scene	Nov 2018
27	Housebreaking	Suspect vs. crime scene	Nov 2018
28	Housebreaking	Suspect vs. crime scene	Nov 2018
29	Housebreaking	Crime scene vs. crime scene	Nov 2018

30	Housebreaking	Crime scene vs. crime scene	Apr 2018
31	Housebreaking	Suspect vs. crime scene	Jan 2019
32	Housebreaking	Suspect vs. crime scene	Mac 2019
33	Housebreaking	Suspect vs. crime scene	Apr 2019
34	Housebreaking	Suspect vs. crime scene	May 2019
35	Housebreaking	Convicted vs. crime scene	May 2019
36	Housebreaking	Convicted vs. crime scene	May 2019
37	Housebreaking	Crime scene vs. crime scene	July 2019
38	Housebreaking	Crime scene vs. crime scene	July 2019
39	Housebreaking	Crime scene vs. crime scene	July 2019
40	Housebreaking	Crime scene vs. crime scene	July 2019
41	Housebreaking	Convicted vs. crime scene	Jan 2020
42	Possession of an unlawful firearm	Suspect vs. crime scene	Oct 2017
43	Possession of an unlawful firearm	Convicted vs. crime scene	May 2019
44	Possession of an unlawful firearm	Suspect vs. crime scene	August 2019
45	Gang-robbery	Drug Dependent vs. crime scene	July 2018
46	Gang-robbery	Suspect vs. crime scene	August 2018
47	Gang-robbery	Suspect vs. crime scene	Apr 2019
48	Gang-robbery	Suspect vs. crime scene	Apr 2019
49	Gang-robbery	Suspect vs. crime scene	Apr 2019
50	Gang-robbery	Convicted vs. crime scene	May 2019
51	Gang-robbery	Suspect vs. crime scene	August 2018
52	Gang-robbery	Suspect vs. crime scene	June 2019
53	Gang-robbery	Suspect vs. crime scene	Sept 2019
54	Gang-robbery	Suspect vs. crime scene	Nov 2015
55	Gang-robbery	Volunteer vs. crime scene	Dec 2015
56	Gang-robbery	Suspect vs. crime scene	Jan 2018
57	Gang-robbery	Convicted vs. crime scene	Oct 2017
58	Murder	Suspect vs. crime scene	Feb 2018
59	Murder	Suspect vs. crime scene	Oct 2017
60	Murder	Suspect vs. crime scene	Feb 2017
61	Murder	Crime scene vs. crime scene	May 2013

62	Murder	Suspect vs. crime scene	August 2019
63	Murder	Convicted vs. crime scene	Dec 2019
64	Identification of unidentified body	Drug Dependent vs. crime scene	Oct 2017
65	Identification of unidentified body	Convicted vs. crime scene	May 2019
66	Identification of unidentified body	Crime scene vs. crime scene	May 2019
67	Identification of unidentified body	Suspect vs. crime scene	Jan 2020
68	Custody / control dangerous drugs	Suspect vs. crime scene	July 2019
69	Carnal intercourse against the order of nature	Convicted vs. crime scene	Nov 2019

vs. refer to a hit between DNA profiles stored in FDDM and those obtained from crime scenes, suspects, detainees, drug dependent, convicted, missing person or volunteers.
*Source:*data were obtained with permission from DNA Databank Division (D13), Criminal Investigation Department, Royal Malaysia Police.

Table S2. List of Y-STR studies in various population groups in Malaysia.

Region	Ethnicity	Sample size (n)	Number of STR loci	PCR Amplification kit	Reference
Peninsular Malaysia (main ethnic group)	Malay	36	12	PowerPlex® Y System (Promega)	[37]
		334	16	AmpFl STR® Yfiler™ kit (Applied Biosystems)	[38]
	Chinese	331	16	AmpFl STR® Yfiler™ kit (Applied Biosystems)	[38]
	Indian	315	16	AmpFl STR® Yfiler™ kit (Applied Biosystems)	[38]
Peninsular Malaysia (Malay sub-ethnic group)	Acheh	7	15	AmpFlSTR® Y-filer™ kit (Applied Biosystems)	[31]
	Champa	13	17		
	Rawa	11	17		
	Kedah	9	17		
	Minangkabau	23	17		
	Bugis	15	17		
	Kelantan	43	17		
	Banjar	18	17		
	Javanese	14	17		
Peninsular Malaysia	Proto-Malay	21	11	NA*	[39]

(Proto Malay)					
East Malaysia - Borneo (Sarawak / Sabah ethnic group)	Iban	103<br>94	17<br>27	AmpFlSTR® Y-filer™ kit (Applied Biosystems)<br>Affymetrix GeneChip Human Mapping 500K Array (Affymetrix)	[40]<br>[12]
	Bidayuh	113	17	AmpFlSTR® Y-filer™ kit (Applied Biosystems)	[40]
	Melanau	104	17	AmpFlSTR® Y-filer™ kit (Applied Biosystems)	[40]
	Kedayan	128	23	PowerPlex® Y23 System (Promega)	[14]
Northern Peninsular Malaysia – Orang Asli	Negrito	33<br>7<br>41	12<br>3<br>11	PowerPlex® Y System (Promega)<br>Q5 High Fidelity Kit (Biolabs)<br>NA*	[37]<br>[41]<br>[39]
	Senoi	14<br>50<br>43	3<br>5<br>11	Q5 High Fidelity Kit (Biolabs)<br>AmpFlSTR® Y-filer™ kit (Applied Biosystems)<br>NA*	[41]<br>[42]<br>[39]

** PCR kit information not available.*

Table S3. List of mtDNA studies in various population groups in Malaysia.

Region	Ethnicity	Sample size (n)	Sequencing platform	Reference
Peninsular Malaysia (main ethnic group)	Malay	14<br>102<br>52<br>15<br>7<br>124<br>248	ABI 380A DNA Synthesizer (Applied Biosystems)<br>ABI PRISM® 310 (Applied Biosystems)<br>ABI Dye Terminator Cycle Sequencing FS (Applied Biosystems)<br>ABI PRISM® 377 (Applied Biosystems)<br>Beckman-Coulter CEQ8000 (Beckman Coulter)<br>ABI 3130 (Applied Biosystems)<br>ABI 3130xl (Applied Biosystems)	[43]<br>[44]<br>[45]<br>[46]<br>[47]<br>[48]<br>[49]
	Chinese	14<br>15	ABI 380A DNA Synthesizer (Applied Biosystems)<br>ABI PRISM® 377 (Applied Biosystems)	[43]<br>[46]
	Indian	15	ABI PRISM® 377 (Applied Biosystems)	[46]

Peninsular Malaysia (others ethnic group)	Punjabi	15	ABI PRISM® 377 (Applied Biosystems)	[46]
Peninsular Malaysia (Malay sub-ethnic group)	Kedah	24	Affymetrix GeneChip Mapping Xba 50 K Arrays (Affymetrix)	[50]
	Minangkabau	20		
	Bugis	14		
	Kelantan	18		
	Javanese	19		
East Malaysia - Borneo (Sarawak / Sabah ethnic group)	Murut	1	ABI 380A DNA Synthesizer (Applied Biosystems)	[43]
		15	ABI PRISM® 377 (Applied Biosystems)	[46]
		2	Illumina HiSeq 2000 (Illumina)	[51]
	Kadazan - Dusun	24	ABI 380A DNA Synthesizer (Applied Biosystems)	[43]
		15	ABI PRISM® 377 (Applied Biosystems)	[46]
		2	Illumina HiSeq 2000 (Illumina)	[51]
	Iban	15	ABI PRISM® 377 (Applied Biosystems)	[46]
	Bajau	15	ABI PRISM® 377 (Applied Biosystems)	[46]
	Bisaya	2	ABI 380A DNA Synthesizer (Applied Biosystems)	[43]
		15	ABI PRISM® 377 (Applied Biosystems)	[46]
	Orang Sungai	15	ABI PRISM® 377 (Applied Biosystems)	[46]
	Sonsogon	1	Illumina HiSeq 2000 (Illumina)	[51]
	Rungus	3	ABI 380A DNA Synthesizer (Applied Biosystems)	[43]
		15	ABI PRISM® 377 (Applied Biosystems)	[46]
		1	Illumina HiSeq 2000 (Illumina)	[51]
	Bidayuh	15	ABI PRISM® 377 (Applied Biosystems)	[46]
		17	ABI 3130xl (Applied Biosystems)	[52]
Peninsular Malaysia – Orang Asli	Semang	52	ABI PRISM® 310 (Applied Biosystems)	[44]
		112	Beckman-Coulter CEQ8000 (Beckman Coulter)	[47]
	Jahai	29	ABI PRISM® 310 (Applied Biosystems)	[44]
		22	ABI 3130xl (Applied Biosystems)	[52]
		50	Affymetrix GeneChip Mapping Xba 50 K Arrays (Affymetrix)	[50]
	Temuan	33	Beckman-Coulter CEQ8000 (Beckman Coulter)	[47]
		8	ABI 3130xl (Applied Biosystems)	[52]

		49	Affymetrix GeneChip Mapping Xba 50 K Arrays (Affymetrix)	[50]
	Semelai	61	Beckman-Coulter CEQ8000 (Beckman Coulter)	[47]
	Jakun	1<br>2	ABI 380A DNA Synthesizer (Applied Biosystems)<br>Beckman-Coulter CEQ8000 (Beckman Coulter)	[43]<br>[47]
	Jeni	2	ABI 380A DNA Synthesizer (Applied Biosystems)	[43]
	Kensiu	29<br>30	ABI PRISM® 310 (Applied Biosystems)<br>Affymetrix GeneChip Mapping Xba 50 K Arrays (Affymetrix)	[44]<br>[50]
	Seletar	21	ABI 3130xl (Applied Biosystems)	[52]
	Temiar	7	ABI 380A DNA Synthesizer (Applied Biosystems)	[43]
	Senoi	52	Beckman-Coulter CEQ8000 (Beckman Coulter)	[47]
	Bateq	2	Illumina HiSeq 2000 (Illumina)	[51]
	Mandriq	2	Illumina HiSeq 2000 (Illumina)	[51]
	Semai	5<br>2	ABI 380A DNA Synthesizer (Applied Biosystems)<br>Illumina HiSeq 2000 (Illumina)	[43]<br>[51]
